# Social-Environmental Injustice and Cancer Screening Prevalence

**DOI:** 10.1001/jamanetworkopen.2024.33724

**Published:** 2024-09-16

**Authors:** Kilan C. Ashad-Bishop, Daniel Wiese, Jordan A. Baeker Bispo, Margaret Katana Ogongo, Farhad Islami, Priti Bandi

**Affiliations:** 1 Department of Environmental Science and Policy, Rosenstiel School of Marine, Atmospheric, and Earth Science, University of Miami, Miami, Florida; 2 Department of Surveillance and Health Equity Science, American Cancer Society, Georgia, Atlanta

## Abstract

This cross-sectional study characterizes the prevalence of cancer screening across geographic clusters of social risk factors, environmental burden, and compounded social-environmental injustice in densely-populated urban areas of the US.

## Introduction

Structural inequity and environmental injustice in the US have patterned cancer disparities, particularly along racial and socioeconomic lines, while reducing the ability of affected communities to avoid these negative health outcomes.^1,2,3^ The relationship between environmental injustice and elevated risk of cancer incidence and mortality is well established,^1,2^ as are relationships between social risk factors and cancer,^3^ but cancer control studies in socially at-risk communities also affected by environmental injustice are less frequent. In this study, we use a novel environmental injustice index to characterize the prevalence of cancer screening across geographic clusters of social risk factors, environmental burden, and compounded social-environmental injustice in 6 densely-populated urban areas.

## Methods

This cross-sectional study followed the Strengthening the Reporting of Observational Studies in Epidemiology (STROBE) reporting guideline. The study was deemed exempt and granted waiver of informed consent by the Morehouse School of Medicine institutional review board, as it used deidentified public-use data. Census-tract level data from 6 counties containing the 6 most populous cities in the US were analyzed. To quantify tract-level exposures of social vulnerability (SV) and environmental burden (EB), we used unweighted percentile ranked sums of the social and environmental indicators from the 2022 release of the Centers for Disease Control and Prevention Environmental Justice Index (EJI). The construction of the EJI has been described in detail.^4^ We used Local Moran I to identify geographic hot spots (*P* < .05) of SV and EB, and defined hot spots of social-environmental injustice (SEI) as hot spots of both SV and EB. Our outcomes were model-based estimates of breast, cervical, and colorectal cancer screening per US Preventive Services Task Force recommendations (eMethods in Supplement 1).^5^ We compared the mean prevalence of cancer screening between hot spots and non–hot spots of SV alone, EB alone, and SEI using 2-sided *t* tests, with significance set at *P* < .05. Analyses were conducted from October 2023 to January 2024 in RStudio version 2023.06.1 + 524 (R Project for Statistical Computing).

## Results

Of the 7791 census tracts analyzed, 410 (5.3%) were identified as SEI hot spots. The highest prevalence of tracts identified as SEI hot spots was among Western counties (Los Angeles County, California; Maricopa County, Arizona) (Figure). No significant differences were observed in the mean population living in SEI, SV, and EB hot spots relative to non–hot spots (Table). SEI hot spots had the lowest mean prevalence of breast (76.6%), cervical (78.4%), and colorectal (51.0%) cancer screening relative to hot spots of SV alone (78.1%, 79.1%, and 53.0%, respectively) and EB alone (78.1%, 81.3%, and 59.0%, respectively) (Table). The prevalence difference for cervical cancer and colorectal cancer screening in SEI hot spots vs non–hot spots was 4.1 (95% CI, 3.6-4.5) and 10.6 (95% CI, 9.8-11.4) percentage points, respectively. For colon cancer and colorectal cancer screening, prevalence differences between hot spots and non–hot spots of SEI and SV alone were similar in magnitude, but larger than those for EB alone (Table). Mean prevalence of cervical cancer and colorectal cancer screening in SEI hot spots in each county was significantly lower than non–hot spots. The prevalence difference for mammography between hot spots (76.6%) and non–hot spots (78.5%) of SEI was 1.9 (95% CI, 1.6-2.3) percentage points, compared with differences of 0.3 (95% CI, 0.1-0.5) percentage points between hot spots and non–hot spots of SV alone and EB alone. Mammography prevalence was significantly lower among SEI hot spots relative to non–hot spots in Los Angeles, Harris, Philadelphia, and Maricopa Counties.

**Figure.**
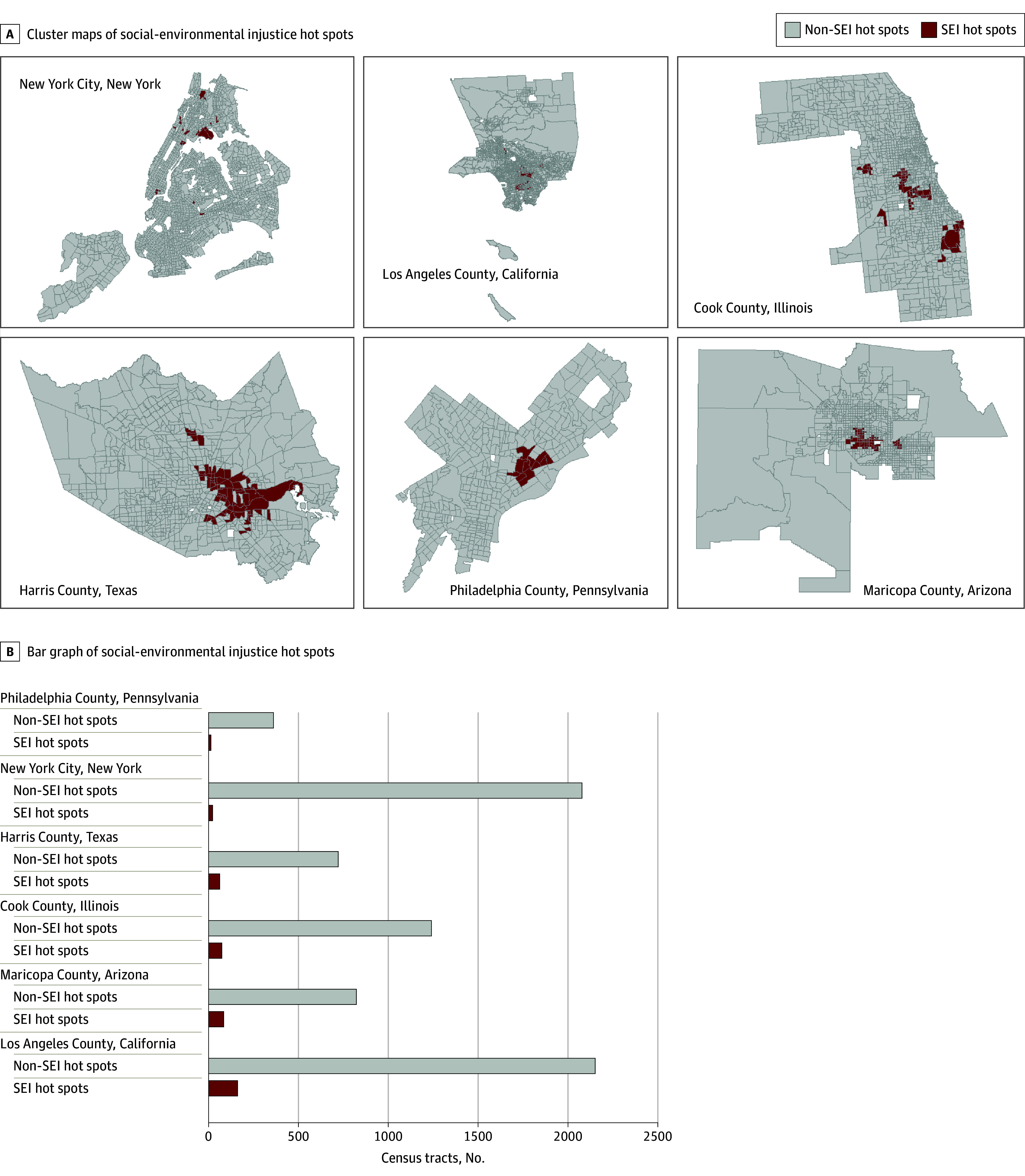
Geographic Distribution of Social-Environmental Injustice (SEI) Hot Spots A, Local Moran I cluster maps of hot spots of SEI in the counties containing the 6 most populous cities in the US. SEI hot spots, determined according to the Environmental Justice Index from the US Centers for Disease Control and Prevention and the Agency for Toxic Substances and Disease Registry, are visualized in dark red. Non–SEI hot spots are visualized in light gray, missing data are visualized in white, and census tract boundaries are visualized in dark gray. B, Bar graph representing number of SEI hot spots (dark red) and non–SEI hot spots (light gray) across the 6 study areas.

**Table.** Prevalence of Cancer Screening in Hot Spots of Social Vulnerability, Environmental Burden, and Social-Environmental Injustice

Characteristic	SEI hot spots (n = 410)	Non–SEI hot spots (n = 7381)	Prevalence difference (95% CI)	*P* value^a^	Hot spot of SV alone (n = 1559)	Non–hot spots of SV alone (n = 6232)	Prevalence difference (95% CI)	*P* value^a^	Hot spots of EB alone (n = 1401)	Non–hot spots of EB alone (n = 6390)	Prevalence difference (95% CI)	*P* value^a^
Population^b^	4140 (1726)	4180 (1892)	NA	.90	4146 (1682)	4186 (1932)	NA	.50	4210 (1855)	4171 (1891)	NA	.30
Cervical cancer screening^b^	78.4 (3.4)	82.5 (4.4)	−4.1 (−4.5 to −3.6)	<.001	79.1 (3.7)	83.0 (4.3)	−3.9 (−4.1 to −3.6)	<.001	81.3 (4.5)	82.5 (4.4)	−1.2 (−1.5 to −1.0)	<.001
New York City, New York	80.8 (5.7)	83.7 (4.7)	NA	.004	81.1 (4.4)	84.0 (4.6)	NA	<.001	84.8 (4.3)	83.2 (4.7)	NA	<.001
Los Angeles County, California	78.8 (3.0)	82.2 (4.5)	NA	<.001	78.2 (3.6)	81.9 (4.4)	NA	<.001	79.8 (3.7)	81.5 (4.7)	NA	<.001
Cook County, Illinois	78.9 (2.6)	82.9 (3.7)	NA	<.001	79.8 (2.7)	82.8 (3.7)	NA	<.001	81.2 (3.8)	82.4 (3.7)	NA	<.001
Harris County, Texas	78.2 (2.5)	82.8 (3.7)	NA	<.001	77.8 (3.1)	82.5 (3.8)	NA	<.001	79.0 (3.1)	81.8 (4.1)	NA	<.001
Philadelphia County, Pennsylvania	79.2 (2.2)	83.5 (4.5)	NA	<.001	79.7 (3.0)	83.3 (4.3)	NA	<.001	81.4 (3.3)	82.9 (4.5)	NA	<.001
Maricopa County, Arizona	77.0 (2.8)	84.5 (2.9)	NA	<.001	78.1 (2.9)	84.1 (3.3)	NA	<.001	78.5 (4.0)	83.7 (3.4)	NA	<.001
Colorectal cancer screening^b^	51 (6)	62 (8)	−10.6 (−11.4 to −9.8)	<.001	53 (6)	63 (7)	−10.6 (−11.0 to −10.2)	<.001	59 (9)	62 (8)	−2.9 (−3.3 to −2.4)	<.001
New York City, New York	56 (6)	62 (7)	NA	<.001	55 (5)	63 (7)	NA	<.001	65 (8)	61 (7)	NA	<.001
Los Angeles County, California	54 (5)	66 (7)	NA	<.001	54 (5)	66 (7)	NA	<.001	59 (7)	64 (8)	NA	<.001
Cook County, Illinois	52 (6)	63 (6)	NA	<.001	53 (6)	63 (6)	NA	<.001	58 (8)	61 (7)	NA	<.001
Harris County, Texas	45 (4)	57 (7)	NA	<.001	45 (6)	56 (8)	NA	<.001	47 (6)	55 (8)	NA	<.001
Philadelphia County, Pennsylvania	53.9 (4.2)	66.4 (6.0)	NA	<.001	54 (6)	66 (6)	NA	<.001	62 (7)	64 (7)	NA	.01
Maricopa County, Arizona	48 (6)	65 (6)	NA	<.001	50 (6)	64 (6)	NA	<.001	53 (8)	63 (7)	NA	<.001
Mammography use^b^	76.6 (3.4)	78.5 (3.4)	−.1.9 (−2.3 to −1.6)	<.001	78.1 (3.7)	78.4 (3.3)	−.0.3 (−0.5 to −0.1)	.30	78.1 (3.6)	78.4 (3.4)	−0.3 (−0.5 to −0.1)	<.001
New York City, New York	81.06 (2.04)	80.56 (3.15)	NA	.30	81.16 (2.20)	80.75 (3.17)	NA	.02	81.45 (3.07)	80.68 (3.01)	NA	<.001
Los Angeles County, California	77.90 (1.55)	78.77 (1.45)	NA	<.001	78.08 (1.59)	78.64 (1.47)	NA	<.001	77.96 (1.45)	78.66 (1.49)	NA	<.001
Cook County, Illinois	79.14 (2.37)	79.41 (2.39)	NA	.30	80.59 (2.35)	79.28 (2.40)	NA	<.001	78.75 (2.35)	79.74 (2.43)	NA	<.001
Harris County, Texas	74.94 (2.33)	76.28 (1.90)	NA	<.001	75.11 (2.39)	76.14 (1.96)	NA	<.001	74.94 (2.21)	76.07 (2.05)	NA	<.001
Philadelphia County, Pennsylvania	75.92 (1.37)	79.31 (2.68)	NA	<.001	78.32 (2.31)	78.69 (2.97)	NA	.20	75.05 (1.32)	79.23 (2.61)	NA	<.001
Maricopa County, Arizona	71.80 (1.47)	73.02 (1.80)	NA	<.001	71.53 (1.41)	73.01 (1.79)	NA	<.001	72.27 (1.69)	72.79 (1.83)	NA	<.001

^a^ Calculated with *t* test.

^b^ Values are mean (SD).

## Discussion

We found that SEI hot spots had the lowest prevalence of all screening modalities, and breast cancer screening prevalence relative to non–hot spots was larger in SEI hot spots than SV-alone or EB-alone hot spots. Identifying areas of greatest SEI can help craft place-based cancer control efforts, which could include identifying place-based structural indicators associated with lower mammography participation alongside direct efforts at bolstering access to breast cancer screening. For colorectal cancer and cervical cancer screening, SEI vs non–hot spot prevalence differences were larger than for breast cancer screening, but largely matched the magnitude of SV-alone differences. This suggests, as previously observed, that SV may drive much of the observed differences among these cancers,^6^ highlighting the need for continued efforts at addressing the place-based social determinants contributing to these disparities. Limitations of this study include cross-sectional ecological study design, use of model-based screening estimates, and restricting the study sample to urban areas due to the sensitivity of hot spot methodology.

## Notes

Supplement 1.
eMethods.

Supplement 2.
Data Sharing Statement
