# Supplementary material for: Social-Environmental Injustice and Cancer Screening Prevalence
Source: JAMA Netw Open. 2024 Sep 16;7(9):e2433724. doi: 10.1001/jamanetworkopen.2024.33724 (PMC11406390; doi:10.1001/jamanetworkopen.2024.33724)
Supplement: Supplement 1. — eMethods. [file jamanetwopen-e2433724-s001.pdf]

Supplemental Online Content

Ashad-Bishop KC, Wiese D, Baeker Bispo JA, Ogongo MK, Islami F, Bandi P. Social-environmental injustice and cancer screening prevalence. *JAMA Netw Open.* 2024;7(9):e2433724. doi:10.1001/jamanetworkopen.2024.33724

eMethods

This supplemental material has been provided by the authors to give readers additional information about their work.

© 2024 Ashad-Bishop KC et al. *JAMA Network Open.*

eMethods

Study Sample

Census-tract level data from the counties (Bronx, Kings, New York, Queens, and Richmond County, NY, Los Angeles County, CA, Cook County, IL, Harris County, TX, Maricopa County, AZ, and Philadelphia County, PA) containing the six most populous cities in the US (New York City, NY, Los Angeles, CA, Chicago, IL, Houston, TX, Phoenix, AZ, and Philadelphia, PA) were analyzed.

Environmental Justice Index

Environmental injustice is the disproportionate exposure of communities of color and the poor to pollution, and its consequent effects on health and the environment, as well as unequal environmental protection and environmental quality provided through laws, regulations, governmental programs, enforcement, and policies. To quantify the exposures of social vulnerability, environmental burden, and social-environmental injustice at the neighborhood level, we utilized the 2022 release of the Environmental Justice Index (EJI) from the Centers for Disease Control and Prevention (CDC). The EJI is a place-based tool that ranks every census tract in the United States based on seventeen indicators of environmental burden and fourteen indicators of social factors that systematically render populations vulnerable to the health impacts of environmental injustice. There are also five indicators of health vulnerability in the Environmental Justice Index, but that module is recommended for exclusion when a health outcome is of interest. Due to a lack of scientific evidence supporting a specific weighting scheme, all modules are weighted equally in calculating the social vulnerability and environmental burden modules. For our analysis, we used the percentile ranked sum for social vulnerability and environmental burden to identify geographic hot spots of each in our six study areas, then computed social-environmental injustice as hotspots of both individual measures.

Social Vulnerability and Environmental Burden Indicators of the Environmental Justice Index	
Domain	Indicator Name
Environmental Burden	Ozone
	PM2.5
	Diesel Particulate Matter
	Air Toxics Cancer Risk
	National Priority List Sites
	Toxic Release Inventory Sites
	Treatment, Storage, and Disposal Sites
	Risk Management Plan Sites
	Coal Mines
	Lead Mines
	Lack of Recreational Park
	Houses Built Pre-1980
	Lack of Walkability
	High-Volume Roads
	Railways

© 2024 Ashad-Bishop KC et al. *JAMA Network Open.*

	Airports
	Impaired Surface Water
Social Vulnerability	Minority Status
	Poverty
	No High School Diploma
	Unemployment
	Housing Tenure
	Housing Burdened Lower-Income Households
	Lack of Health Insurance
	Broadband Access
	Age 65 and Older
	Age 17 and Younger
	Civilian with Disability
	Speaks English "Less than Well"
	Mobile Homes
	Group Quarters

CDC PLACES

The outcomes of this study were census tract-level prevalence of USPSTF guideline-concordant, up-to-date breast (mammography), cervical, and colorectal screenings from the CDC PLACES dataset. These are the only screen-detectable cancers included in the CDC PLACES dataset. PLACES provides small area estimations of 29 chronic disease health outcomes and health behaviors based on Behavioral Risk Factor Surveillance System (BRFSS) data and US Census data. We utilized the 2020 PLACES dataset, which corresponds to survey responses in the 2018 BRFSS.

© 2024 Ashad-Bishop KC et al. *JAMA Network Open.*
