# Supplementary material for: Social-Environmental Injustice and Cancer Screening Prevalence
Source: JAMA Netw Open. 2024 Sep 16;7(9):e2433724. doi: 10.1001/jamanetworkopen.2024.33724 (PMC11406390; doi:10.1001/jamanetworkopen.2024.33724)
Supplement: Supplement 2. — Data Sharing Statement [file jamanetwopen-e2433724-s002.pdf]

Data Sharing Statement

Ashad-Bishop. Social-Environmental Injustice and Cancer Screening Prevalence. *JAMA Netw Open*. Published September 16, 2024. doi:10.1001/jamanetworkopen.2024.33724

Data
Data available: No

Additional Information
Explanation for why data not available: All data is publicly available via the Centers for Disease Control and Prevention website.
